# Streamlined preparation of genomic DNA in agarose plugs for pulsed-field gel electrophoresis

**DOI:** 10.14440/jbm.2018.218

**Published:** 2018-03-09

**Authors:** Linda Hicks, Charlotte M. van der Graaf, Jacob Childress, Emily Cook, Karen Schmidt, Frank Rosenzweig, Eugene Kroll

**Affiliations:** Division of Biological Sciences, University of Montana, Missoula, MT 59812, USA

**Keywords:** pulsed-field gel electrophoresis, deep-well 48-well plate, chromosomal DNA preparation, DNA plugs, karyotyping, genomic fingerprinting

## Abstract

Genome analysis using pulsed-field gel electrophoresis (PFGE) has been used in applications ranging from typing bacterial strains to radiobiology to cancer research. While methods for running PFGE have been significantly improved since its invention, the method for preparing chromosomal DNA itself has remained essentially unchanged. This limits the applicability of PFGE, especially when analyses require many samples. We have streamlined sample preparation for routine applications of PFGE through the use of deep-well 48-well plates. Besides saving time, our protocol has the added advantage of reducing the volume of expensive reagents. Our improved protocol enables us to reduce throughput time and simplify the procedure, facilitating wider application of PFGE-based analyses in the laboratory.

## BACKGROUND

Pulsed-field gel electrophoresis (PFGE) enables large DNA molecules, including whole chromosomes, to be easily separated and visualized [1,2]. PFGE is routinely used in the clinical laboratory to fingerprint restriction-fragmented bacterial genomes, especially those of epidemiological interest [3,4], and to karyotype eukaryotic genomes for genetic screening [5]. In addition, PFGE is a powerful research tool to study DNA damage and repair during tumorigenesis [6] and following radiation-induced damage [7]. Since its invention more than 30 years ago, the hardware for performing PFGE and the methods for carrying out image analysis of electropherograms have markedly improved [8,9]. However, the laborious method of preparing samples for PFGE has remained essentially the same. Agarose plugs are individually processed in separate tubes or plug molds, then inserted in gel wells one-by-one. The established method for sample preparation is time-consuming, has the potential for sample mix-up and is resistant to scale-up.

Here we present a streamlined method for preparation of chromosomal DNA for pulsed-field gel electrophoreses. The experiment is performed in a single, reusable vessel from start to finish, with up to 48 samples prepared simultaneously. Handling time is reduced six-fold and the amount of required reagents is scaled down by four-fold. The main distinction between our method and standard PFGE preparation is in using one vessel (a single well on a 48 deep-well plate) to grow cultures, prepare agarose plugs, process plugs in batches, and load the gel, without having to handle tubes and DNA plugs separately or to insert individual plugs into gel wells. While future DNA sequencing-based methods may eventually displace PFGE, its simple analytical pipeline, relatively low cost of apparatus and reagents and the ease of training new personnel is likely to keep it a viable genotyping tool for many years to come.

While we used our method with several yeast species, we also adapted this method to accommodate the restriction step necessary for circular bacterial genomes (data not shown). Indeed, PFGE continues to maintain its status as a standard tool for genotyping both opportunistic and frank bacterial pathogens by profiling their restriction-fragmented genomes [10]. Among the latter are pathogens of epidemiological interest such as *Vibrio cholera, Salmonella enterica* [11], MRSA [12] and *Pseudomonas aeruginosa* [13]. We trust that our novel method, used in conjunction with such techniques as multi-locus sequence typing and antimicrobial resistance pattern analysis, has broad potential for application in epidemiological research.

## MATERIALS

### Reagents

- Zymolyase 20T (Sunrise Science Cat, San Diego, CA, cat. #NO766391)
- Proteinase K (VWR, Radnor, PA, cat. #97062-238)
- Low-melting point (LMP) agarose (Invitrogen, Carlsbad, CA, Cat #16520)
- N-Lauroylsarcosine sodium salt (Sigma-Aldrich, St-Louis, MO, cat. #L5125)
- EDTA (Thermo Fisher Scientific, Inc. Hampton, NH, cat. #BP-120)
- Tris-HCl, pH 7.4 (Lonza, Conley, GA, cat. #51237)
- Pulse Field Certified Agarose (Bio-Rad, Hercules, CA, cat. #1620137)
- Ethidium bromide, 10 mg/ml (Teknova. Inc. Hollister, CA, cat. #E3050)
- Tris Base (Thermo Fisher Scientific, Inc., cat. #BP152-1)
- Boric Acid (Thermo Fisher Scientific, Inc., cat. #A74-1)

### Recipes

- T10E50 (10 mM Tris-HCl, pH 7.4, 50 mM EDTA): Mix 10 ml of 1 M Tris-HCl, pH7.4 with 100 ml 0.5 M EDTA in 890 ml dH_2_O.
- T10E500 (10 mM Tris-HCl, pH 7.4, 500 mM EDTA): Mix 1 ml of 1 M Tris-HCl, pH7.4 with 99 ml 0.5 M EDTA.
- 1% LMP agarose: Mix 25 ml 0.5 M EDTA with 75 ml dH_2_O, add 1 g of low-melting point agarose and heat until agarose is completely dissolved and in solution.
- Zymolyase stock solution (20 mg/ml): Add 20 mg to 1 ml 50% glycerol. Store at **–**20°C.
- Zymolyase working solution: Add 30 μl of Zymolyase stock solution (20 mg/ml) to 3 ml of T10E50 (this is enough for 30 wells and can be adjusted accordingly). Make working digestion buffer immediately prior to use, discard unused portion.
- Sarkosyl stock solution: Dissolve 1 g of N-Lauroylsarcosine sodium salt in 100 ml T10E500.
- Proteinase K stock solution (20 mg/ml): Add 20 mg proteinase K to 1 ml 50% glycerol. Store at **–**20°C.
- Proteinase K/sarkosyl working solution: Add 30 μl of proteinase K (20 mg/ml) stock solution to 3 ml of sarkosyl stock solution (this is enough for 30 wells and can be adjusted accordingly), make immediately prior to use and discard unused portion.
- 10× TBE: Add 108 g Tris Base, 55 g boric acid, 40 ml 0.5 M EDTA to a final volume of 1 L dH_2_O.
- 0.5× TBE: Mix 100 ml of 10× TBE with 1900 ml of dH_2_O.

### Equipment

- Reusable 48 well plate-5 ml rectangle well, pyramid bottom clear polypropylene (E&K Scientific, Santa Clara, CA, cat. #EK-2044)
- Breathe-easy gas permeable sealing membrane (RPI Corp, Mount Prospect, IL, cat. #248738)
- 30°C Shaker/incubator (New Brunswick Scientific, Edison, NJ, Model G25)
- Centrifuge with plate rotor (*e.g.* Beckman, Brea, CA, Allegra 25R Centrifuge, S-5700 rotor)
- Repeater pipette, capable of delivering 100–1000 μl (*e.g.* Eppendorf Repeater Plus)
- Vortex with plate adaptor (Benchmark, cat. #AM10014)
- Water bath at 37°C, 55°C and 75°C
- Vacuum aspirator (2 L filter flask + vacuum tubing and pipette tip)
- Rocking platform (Fisher Scientific, Model #88861045)
- 3M^®^ book tape or similar packing tape (3” wide)
- CHEF-DR II system (Bio-Rad)

## PROCEDURE

### Inoculation and growth • TIME 10 min

1. Add 1 ml of liquid growth medium to each well of an E&K Scientific 48 well plate.
  **NOTE:** E&K 48-well plates are made of polypropylene making them autoclavable and re-usable. Prior to inoculation, ensure that plates are clean and sterile.
2. For each sample, pick a small amount of cells from an isolated colony with a sterile toothpick and insert into medium-filled well. Remove toothpicks.
3. Cover plate with Breathe-easy gas permeable sealing membrane.
4. Grow cells at 30°C with shaking overnight.
5. Measure optical absorbance at A_600_ for all cultures. Cell amount can be varied to achieve A_600_ between 2 and 4.
  **CRITICAL STEP:** In order to ensure optimal chromosomal preparation for each sample, absorbance should neither be too high nor too low. Too many cells can cause smearing in the lanes, while too few can result in faint chromosomal bands.

### Washing, preparation of agarose plugs and digestion of cells • TIME 35 min (excluding 4 h to overnight incubation)

***6.*** Pellet cells from overnight growth. Centrifuge cells in the plate using a plate rotor, 5 min at 5000 rpm.
  **NOTE:** All centrifugation steps are done at room temperature (~25°C).
***7.*** Remove supernatant by quickly inverting the plate, or with aspirator.
  **TIP:** After inverting plate, remove excess liquid with a forceful inverted flick.
***8.*** Wash cells by adding 1 ml of dH_2_O to each well.
***9.*** Resuspend cells by mixing on vortex for ~1 min using a plate adaptor.
  **TIP:** After 30 s, turn plate 180°C to ensure even mixing.
***10.*** Centrifuge plate for 5 min at 5000 rpm at room temperature.
***11.*** Remove supernatant by briefly inverting the plate and flicking as above, or with aspirator.
***12.*** Wash cells by adding 1 ml of T10E50 to each well then resuspend, as in steps 8 and 9.
***13.*** Centrifuge plate for 5 min at 5000 rpm.
***14.*** Remove supernatant by briefly inverting the plate and flicking as above, or use aspirator.
***15.*** Prepare 1% LMP agarose by adding 0.5 g LMP agarose to 50 ml of 0.125 M EDTA and carefully melting to homogeneity on low setting in microwave.
  **CRITICAL STEP:** Ensure that agarose is completely melted before cooling to 55°C, and use within 3 min of preparation.
  **TIP:** Prepare Zymolyase working solution while agarose cools. Add 30 μl of Zymolyase stock solution (20 mg/ml) to 3 ml of T10E50 (this is enough for 30 wells and can be adjusted accordingly).
***16.*** Quickly add 100 μl of Zymolyase working solution per well using repeater pipette.
  **CRITICAL STEP:** This step should not take longer than 1 min.
  **TIP:** Addition of 1% LMP to wells can be done in several stages to ensure that the agarose does not solidify.
***17.*** Add 150 μl% LMP agarose per well with repeater pipette. Immediately after the addition of LMP agarose, resuspend the cells for 1 min using a vortex with plate adapter.
  **CRITICAL STEP:** It is critical to vortex immediately, and to verify that all cultures have been completely resuspended by visually examining the plate. If cultures have not been completely resuspended, cell pellets will be visible in the bottom of the well and vortex time should be increased.
***18.*** Place the plate on ice for 5 min to allow agarose to set. Agarose plugs will solidify and adhere to the bottom.
***19.*** Add 150 μl of T10E500 per well and cover the entire plate with 3M^®^ book tape.
***20.*** Incubate for at least 4 h in a 37°C water bath.
  **NOTE:** The plate floats on the surface of the water and does not require a support.
  **TIP:** Incubation at 37°C can be increased from 4 h to overnight if needed.
  **NOTE:** After digestion with Zymolyase, agarose plugs should become transparent.

### Adding proteinase K • TIME 5 min (excluding overnight incubation)

***21.*** Add 100 μl of proteinase K/sarkosyl working solution to each well.
  **NOTE:** Add proteinase K/Sarkosyl working solution directly to plugs using a repeater pipette.
***22.*** Re-seal the plate with fresh 3M^®^ book tape.
***23.*** Incubate overnight in a 55°C water bath.

### Plug washing • TIME 20 min hands-on

***24.*** Add 1 ml of room temperature dH_2_O to each well/plug and immediately aspirate.
  **CRITICAL STEP:** Take care not to touch agarose plugs with aspirator tip as they are fragile and can easily be lost.
  **TIP:** To ensure liquid is completely removed and plugs are not disturbed, tip the 48-well plate at an angle and slowly remove liquid from top to bottom, with a P20 pipette tip attached to the aspirator.
***25.*** Wash plugs by adding 1 ml T10E50 to each well then rock gently on rocking platform for 20 min.
***26.*** Remove T10E50 with aspirator, as above.
***27.*** Wash plugs by adding 1 ml fresh T10E50 to each well then rock gently on rocking platform for 40 min.
***28.*** Remove T10E50 with aspirator, as above.
***29.*** Wash plugs by adding 1 ml fresh T10E50 to each well then rock gently on rocking platform for 60 min.
  **TIP:** During the last wash, prepare the gel for PFGE according to manufacturer requirements. Pre-warm water bath to 75°C.

### Preparation of PFG and loading plugs • TIME 15 min hands-on

***30.*** Weigh out 1.5 g Pulsed Field Certified agarose in a 250 ml flask, and add to 150 ml of 0.5× TBE. Microwave, checking frequently for boiling, and confirm that agarose is completely melted.
  **NOTE:** Prepare 2 L of 0.5× TBE from 10× TBE stock, use 150 ml for gel and remainder for gel running buffer.
***31.*** Prepare gel frame on level surface, with comb positioned evenly. Comb teeth approximately 1 mm from gel frame base.
***32.*** When agarose has cooled to 55°C, slowly pour into gel frame and let cool for 30 min.
  **NOTE:** Pour slowly and evenly to avoid formation of bubbles. If bubbles are present, a small sterile pipette tip can be used to remove them.
***32.*** Pour remaining 0.5× TBE into gel rig chamber and cool to 15°C.
***34.*** From the 48-well plate, remove T10E50 from wells with aspirator, ensuring that all droplets are removed from the sides of wells.
***35.*** Incubate the plate in a 75°C water bath for 5 min.
  **TIP:** Make sure the agarose plugs have melted by gently shaking the plate.
***36.*** Transfer the multi-well plate from 75°C bath to 70°C heat block.
  **CRITICAL STEP:** Agarose plugs should not be incubated longer than 5 min at 75°C. Longer incubations will degrade DNA fragments. Allowing the plate to sit at 70°C will give more time to load the plugs onto the gel before the agarose solidifies, and will help reduce DNA degradation.
***37.*** Clip off 20 μl pipette tip with razor blade. Tip orifice diameter should be ~1 mm. One tip can be used for loading all plugs into gel.
  **NOTE:** A larger diameter orifice tip will ensure that chromosomal DNA is not sheared during loading.
***38.*** Carefully remove comb from gel.
***39.*** Load 20 μl of each liquid agarose plug sample with a clipped-off pipette tip.
  **TIP:** Rinse tip with 70°C water between loadings.
  **NOTE:** Volume of agarose loaded into each well can be increased or decreased to optimize the concentration of DNA in each well.
***40.*** After all the samples are loaded, immediately remove plate from heat block, and place gel into cooled buffer.
***41.*** Let plugs in 48-well plate solidify then overlay each plug with 250–500 μl of T10E500 for long term storage.
***42.*** Cover plate with 3M^®^ tape and store at 4°C, for up to 6 months.
  **NOTE:** Plugs can be re-melted and loaded up to 4 times.
***43.*** Run gel at optimized parameters, for instance for *Saccharomyces*, 6 V, 60–100 s switch time, for 30 h at 15°C.
***44.*** After run, remove gel and place in container with 1 L 0.5× TBE buffer for staining.
***45.*** Add 10 μl of 10 mg/ml ethidium bromide. Mix gently on rocking platform for 30 min.
***46.*** Visualize chromosomes on UV transilluminator.
  **TIP:** Running buffer can be reused for making staining solution. Gel can be stained overnight at 4°C to increase band intensity. Destaining usually is not necessary.

## ANTICIPATED RESULTS

**Figure 1.**
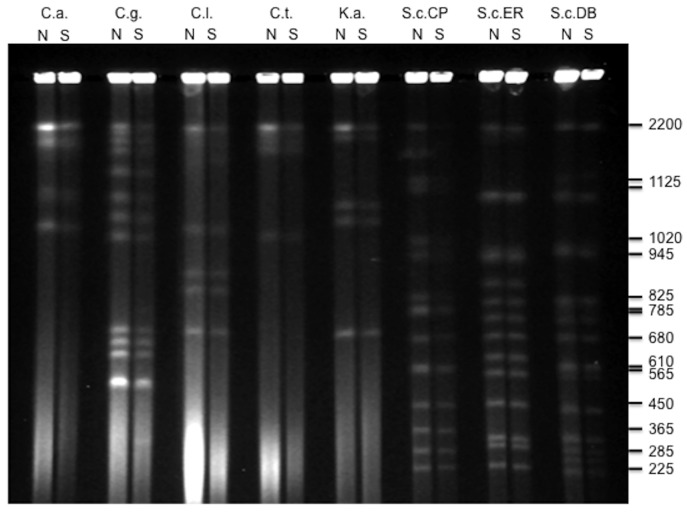
Comparison of yeast chromosomal DNA plugs prepared by standard and new methods. Several yeast species *Candida albicans* (C.a.), *C. glabrata* (C.g.), *C. lusitaniae* (C.l.), *C. tropicalis* (C.t), *Kloeckera apis* (K.a.) and *Saccharomyces cerevisiae* strains CEN.PK (S.c.PK), Ethanol Red (S.c.ER) and DB146 (S.c.DB) were prepared for PFGE using the new method (N) and the standard method (S), and run using conditions optimized for the *S. cerevisiae* chromosomes. Note that while these conditions are not optimal for *Candida sp*. and *Kloeckera* chromosomes, both methods allow yeast chromosomes to enter the gel and separate. Size marker shows approximate chromosome sizes in Kb for *S. cerevisiae* CEN.PK.

This method greatly reduces the time required to prepare and load samples, and also cuts back on plasticware usage and volume of reagents. While the basic chemistry of the preparation technique remains unchanged, sample handling time is reduced by six-fold to approximately one hour. We routinely apply this streamlined method to karyotyping yeast genomes and have demonstrated that this method can be easily adapted to karyotyping a wide range of yeast species. To improve resolution of PFGE bands in other strains or species, further optimization may be required, such as modification of incubation times, adjustment of enzyme concentrations, and/or adjustment of electrophoresis run and switch times. To validate the protocol in yeasts we karyotyped several strains of the budding yeast *Saccharomyces cerevisiae* ((DB146 [14], CEN.PK [15] and Ethanol Red (LeSaffre, France)), the opportunistic pathogen *Candida albicans* (ATCC 90235), *C. glabrata* (ATCC 2001), *C. lusitaniae* (ATCC 34449), *C. tropicalis* (ATCC 14246), and the chocolate fermenter *Kloeckera apis*. (ATCC 32875). We successfully resolved whole chromosomes for each species with accuracy and reproducibility comparable to, or better than that achieved using the standard method (**Fig. 1**).

Increased throughput is achieved by parallel processing of samples. In preparing chromosome-containing agarose plugs the most time-consuming operations are handling of liquid cultures, processing of plugs one-by-one, and loading plugs on the gel. Here we report a protocol that streamlines this process. We carry out culture growth and sample processing in the same vessel (a single well of a multi-well plate), and load melted plugs using a micropipette. Up to 48 cultures can be processed in parallel. Throughput is therefore limited only by the number of wells on a gel. Our method uses the same chemistry as the conventional protocol [2], but significantly reduces handling time required to process and load chromosome plugs. Using the conventional method, handling time is approximately six hours per 30-plug preparation (not counting incubation time); our method reduces the handling time by six-fold to one hour total. The 48 deep-well plate is reusable, obviating the need for disposable tubes and plug molds. Also, the quantity of reagents used per sample is scaled down four-fold, creating additional savings. Finally, loading time and effort, which previously required tedious insertion of pre-formed agarose plugs into gel wells, is now also reduced. In our experience, a 30-well gel can be loaded in less than 10 min. Furthermore, two plates (up to 96 samples) can be processed simultaneously.

## TROUBLESHOOTING

Possible problems and their troubleshooting solutions are listed in **Table 1**.

## Figures and Tables

**Table 1.** Troubleshooting.

Step	Problems	Causes	Suggestions
5	Chromosome plug not clear in step 21	Too many cells Bad Zymolyase	Experiment with fewer cells Confirm digest under microscope
6	Loss of cells during washing	Not enough spinning time	Centrifuge for longer
16	Less DNA than expected in gel matrix	Incomplete cell lysis	Switch to a different cell wall degrading enzyme
17, 18, 32	Smearing of DNA bands	LMP agarose not mixed well with cell suspension Incomplete digestion by proteinase K Excess heating before gel loading	Shorten time between the addition of LMP agarose and mixing Increase the time or concentration of proteinase K Ensure that plugs do not spend longer than 5 min at 75°C
7, 11, 14, 21, 23, 25, 31	Loss of plug	Pressure of aspirator too high Aspirator too close to the plug LMP agarose did not set before addition of buffer	Reduce vacuum pressure of aspirator or switch to manual pipette Position aspirator near the wall of the well Make sure LMP agarose is set before adding buffer
